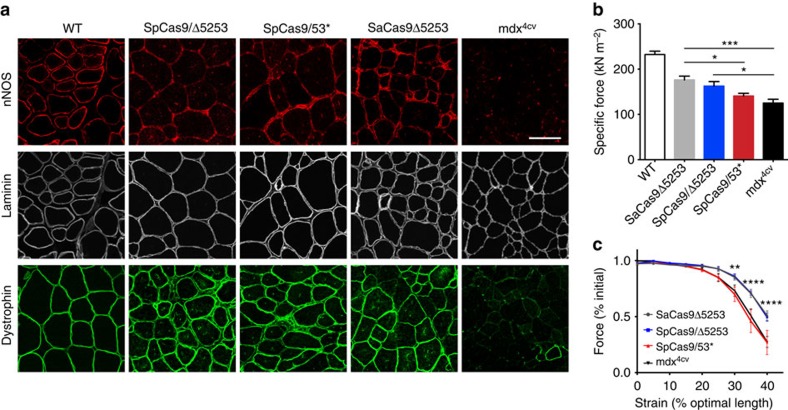# Corrigendum: Muscle-specific CRISPR/Cas9 dystrophin gene editing ameliorates pathophysiology in a mouse model for Duchenne muscular dystrophy

**DOI:** 10.1038/ncomms16007

**Published:** 2017-06-23

**Authors:** Niclas E. Bengtsson, John K. Hall, Guy L. Odom, Michael P. Phelps, Colin R. Andrus, R. David Hawkins, Stephen D. Hauschka, Joel R. Chamberlain, Jeffrey S. Chamberlain

Nature Communications 8: Article number: 14454; DOI: 10.1038/ncomms14454 (2017); Published: 02 14 2017; Updated: 06 23 2017

This Article contains an error in Fig. 4, for which we apologize. In panel a, the image reporting dystrophin labelling following SaCas9Δ5253 treatment was inadvertently duplicated from the corresponding image following SpCas9/Δ5253 treatment. The correct version of this figure appears below as Fig. 1. The raw data associated with this experiment are provided as a separate Supplementary Data file.

## Supplementary Material

Supplementary Data 1

## Figures and Tables

**Figure 1:**