# Supplementary material for: Muscle-specific CRISPR/Cas9 dystrophin gene editing ameliorates pathophysiology in a mouse model for Duchenne muscular dystrophy
Source: Nat Commun. 2017 Feb 14;8:14454. doi: 10.1038/ncomms14454 (PMC5316861; doi:10.1038/ncomms14454)
Supplement: Supplementary Information — Supplementary Figures and Supplementary Tables. [file ncomms14454-s1.pdf]

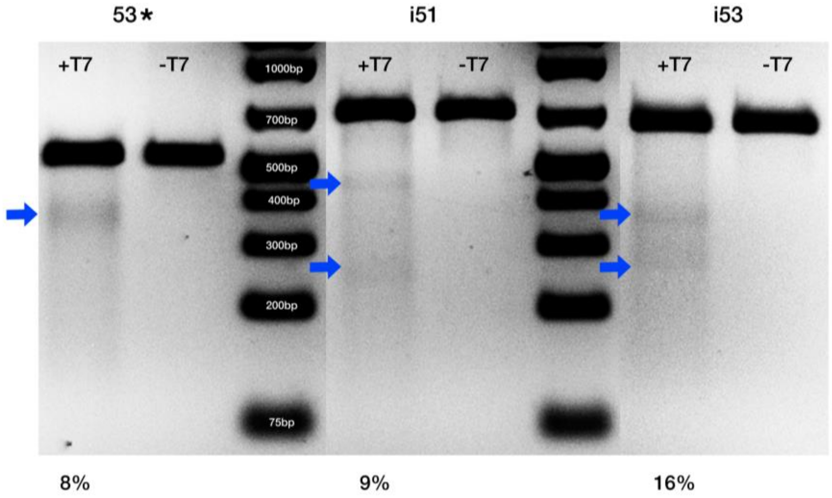


**Supplementary Figure 1: *In vitro* validation of targeting constructs.** *In vitro* editing efficiency at target sites within exon 53 (53*, strategy 2) as well as within introns 51 and 53 (Δ5253, strategy 1) was determined via the T7 endonuclease 1 assay following nuclease- and targeting construct electroporation into primary dermal fibroblasts isolated from *mdx*$^{4cv}$ mice. For strategy 2, the two target sites within exon 53 were analyzed together due to their close proximity to each other. Efficiency estimated via densitometry measurements of unique cleavage bands.

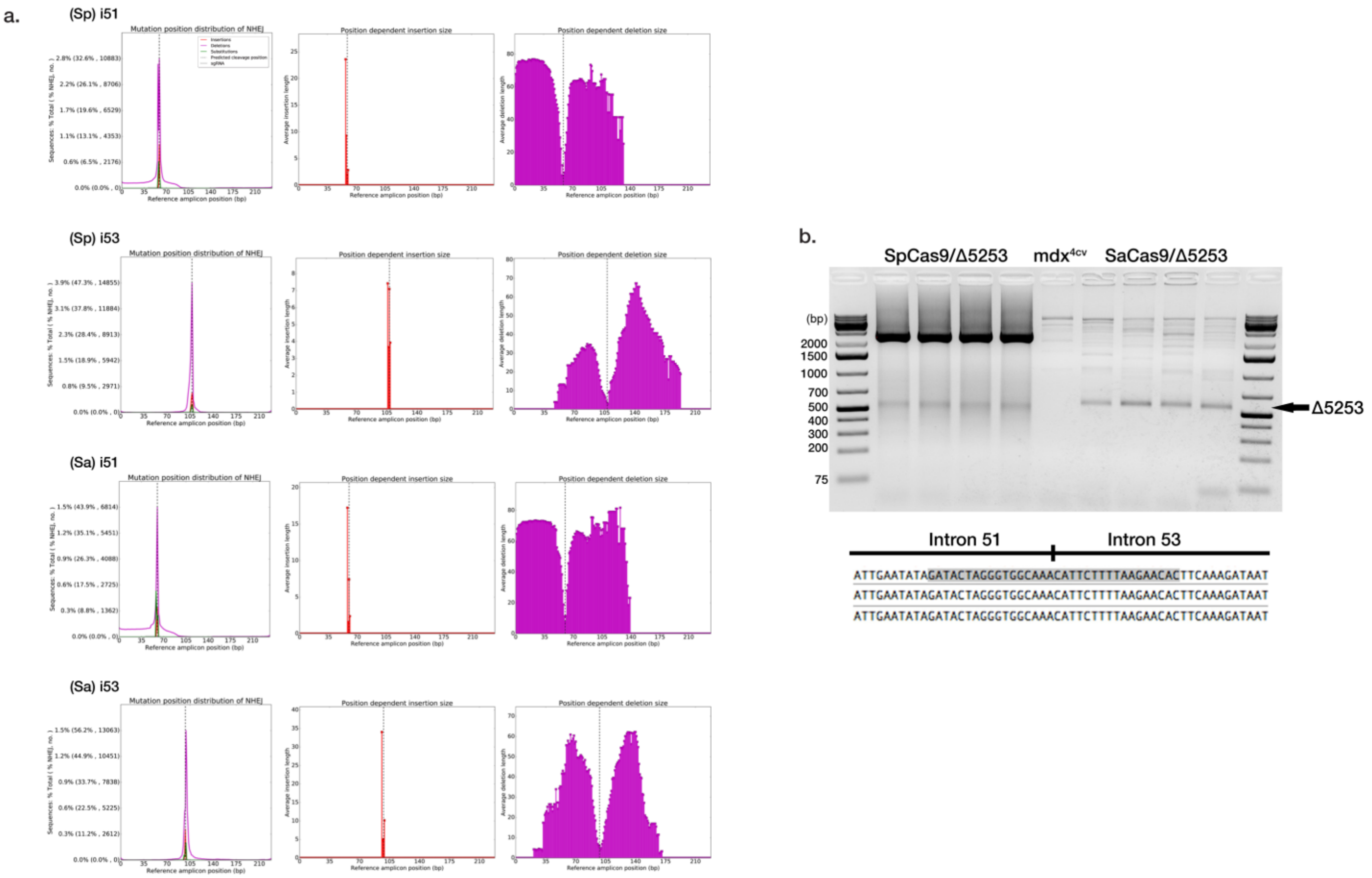


**Supplementary Figure 2: Analyses of gene editing efficiency for strategy 1.** Graphs generated by the CRISPResso software pipeline during genomic deep sequencing analysis of PCR amplicons generated across the target sites within intron 51 (i51) and intron 53 (i53) for strategy 1 (Δ5253). (**a**) Shown are the percentages of insertions, deletions and substitutions, resulting from NHEJ events, for each nucleotide position across the PCR amplicon (left panels). The y-axis represents % total genomes or (% genomes, number of genomes exhibiting NHEJ). Also shown is the average insertion size (center panels) and average deletion size (right panels**)** at each nucleotide position across the PCR amplicons. Dotted lines represent predicted Cas9 cleavage sites. (**b**) PCR across the ~45 kb region targeted for deletion on DNA isolated from TAs treated with strategy 1 (Δ5253, n=4) shows the presence of a unique product (arrow) that by subsequent cloning and sequencing was determined to correlate to a merging of introns 51 and 53 as predicted.

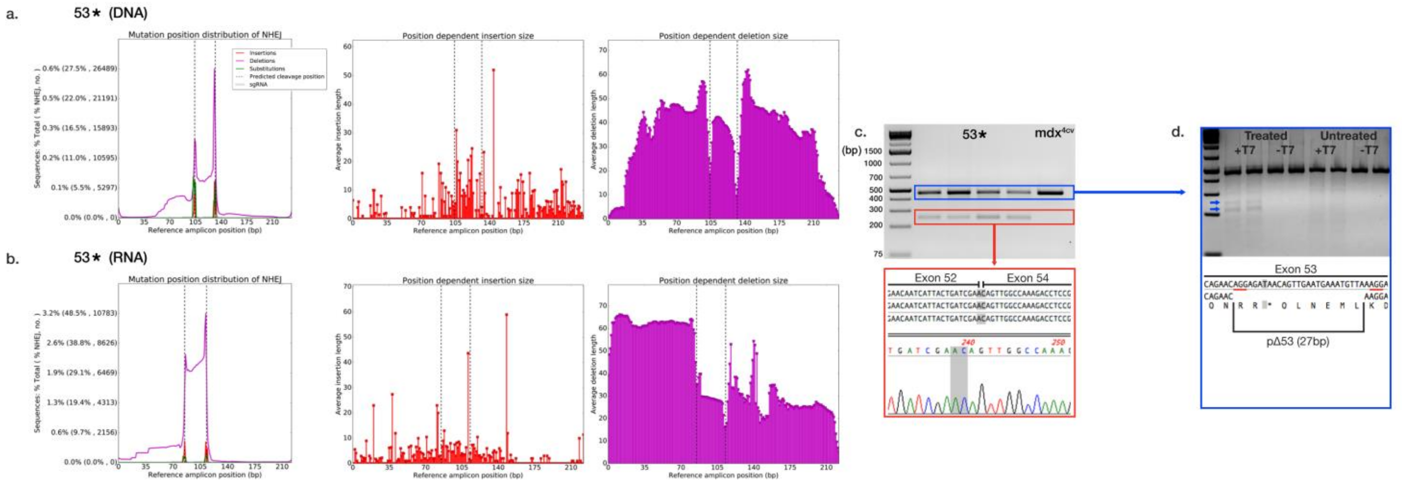


**Supplementary Figure 3: Analyses of gene editing efficiency for strategy 2.** Graphs generated during CRISPResso analysis for strategy 2 (53) during deep sequencing analysis of pooled PCR (**a, n=5**), and RT-PCR (**b, n=4**) amplicons generated across the target sites within exon 53 for strategy 2 (53*). Shown are the percentages of insertions, deletions and substitutions, resulting from NHEJ events, for each nucleotide position across the amplicons (left panels). The y-axis represents % total genomes or (% genomes, number of genomes exhibiting NHEJ). Also shown is the average insertion size (center panels) and average deletion size (right panels**)** at each nucleotide position across the amplicons. Dotted lines represent predicted Cas9 cleavage sites. (**c**) RT-PCR of target region transcripts isolated from TAs treated with strategy 2 (53*, n=4) shows the presence of a shorter product (red box) making up approximately 20% of total transcripts (based on image densitometry). Subsequent cloning and sequencing revealed that this product corresponded to out-of-frame transcripts lacking the sequences encoded on exon 53. (**d**) T7 endonuclease 1 digestion of the predominant top RT-PCR product from muscles treated with strategy 2 (blue box in c) indicates the presence of unique transcripts, making up approximately 11.2% of the analyzed RT-PCR product based on image densitometry (Top, blue arrows). Also shown is the DNA sequence of one clone of the RT-PCR products, revealing an in-frame transcript where the nonsense mutation was removed by a 27 bp in-frame deletion (bottom).

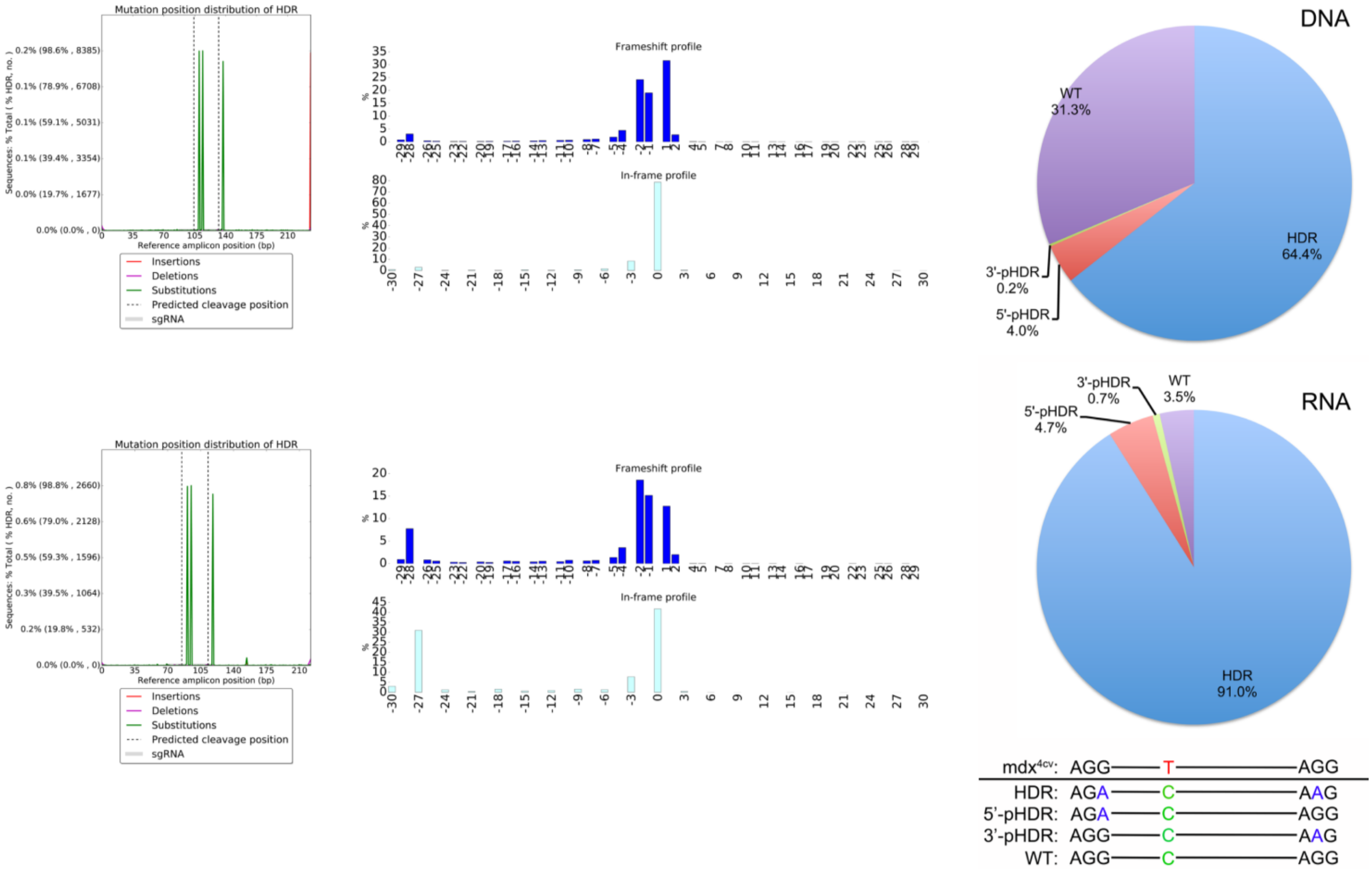


**Supplementary Figure 4: HDR and reading frame analyses for strategy 2.** Graphical representation of HDR detection, reading frame analysis and distribution of HDR genotypes for exon 53 based on deep sequencing of pooled PCR amplicons generated from genomic DNA **(top, n=5)** or transcripts (cDNA) **(bottom, n=4)** isolated from muscles treated with strategy 2 (53*). (**Left panels**) Shown are the percentages of HDR-derived nucleotide substitutions for each position across the amplicons, as generated by the CRISPResso software pipeline. The y-axis represents % total genomes or (% genomes, number of genomes exhibiting HDR). The graph demonstrates nucleotide substitutions at sites corresponding to the two silent PAM site mutations (G to A) and at the site of the *mdx*$^{4cv}$ C to T point mutation. Dotted lines represent predicted Cas9 cleavage sites. (**Middle panels**) Shown are the size distributions of frameshift and in-frame reads, as generated by the CRISPResso (middle panels). The y-axis represents % of frameshift or in-frame reads while the x-axis represent the size of the corresponding deletions, insertions and substitutions. (**Right panels**) Shown are the genotypes and corresponding frequencies of HDR events resulting in the substitution of the *mdx*$^{4cv}$ T mutation (red) to the WT C nucleotide (green). Genotypes resulting from successful HDR consisted of substitutions for: the complete HDR template (HDR), a partial HDR template from the 5'- or 3' ends (5'/3'-pHDR) and substitution of T to C without the PAM site mutations (WT). The silent PAM site mutations are depicted in blue. Of note, WT genotypes may in fact contain a large proportion of background reads, based on the level of reads observed with random single nucleotide substitutions and in untreated control RNA samples (supplemental tables 2-3). The remaining HDR reads containing 2 or 3 defined HDR specific nucleotide substitutions appear highly specific as these reads exhibit close to zero prevalence in untreated RNA samples (supplementary table 3).

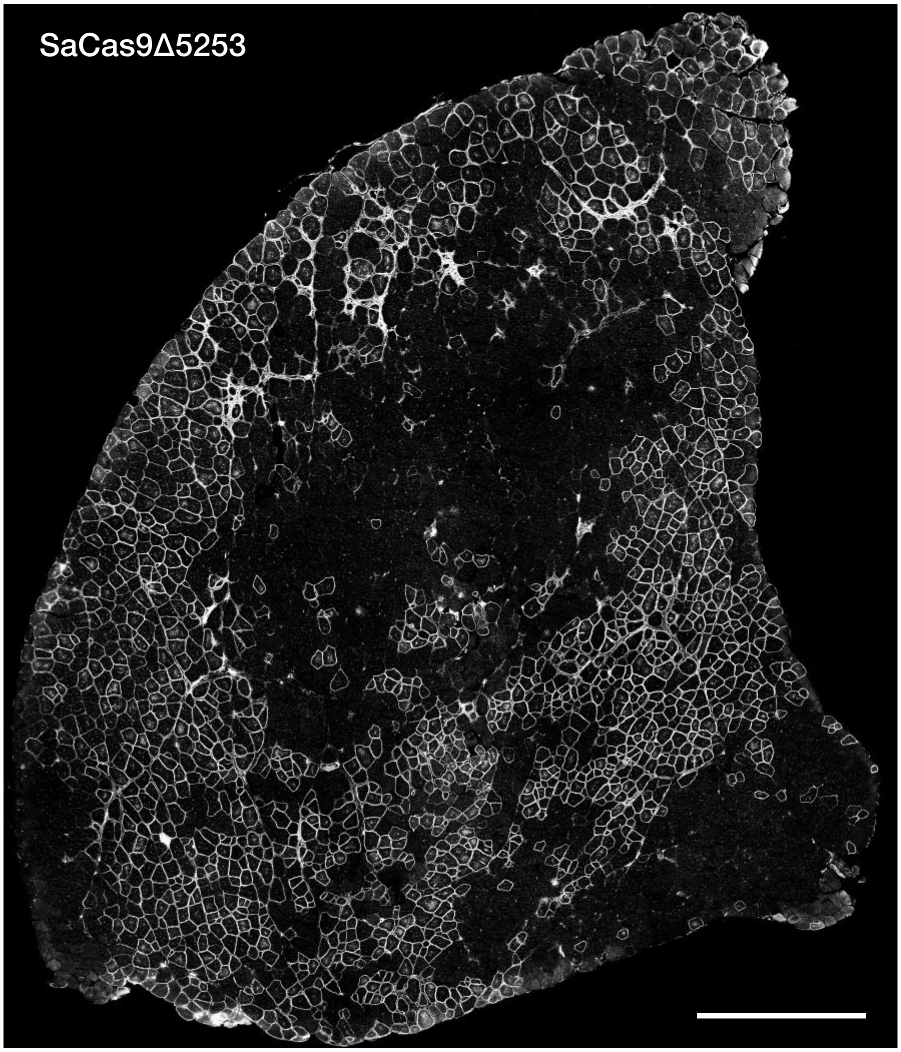


**Supplementary Figure 5: IF analysis for single vector approach in strategy 1.** Dystrophin expression (white) detected in treated TA muscles injected with the single AAV6/SaCas9Δ5253 vector (n=4), analyzed at 4 weeks post-transduction (scale bar = 1000 µm).

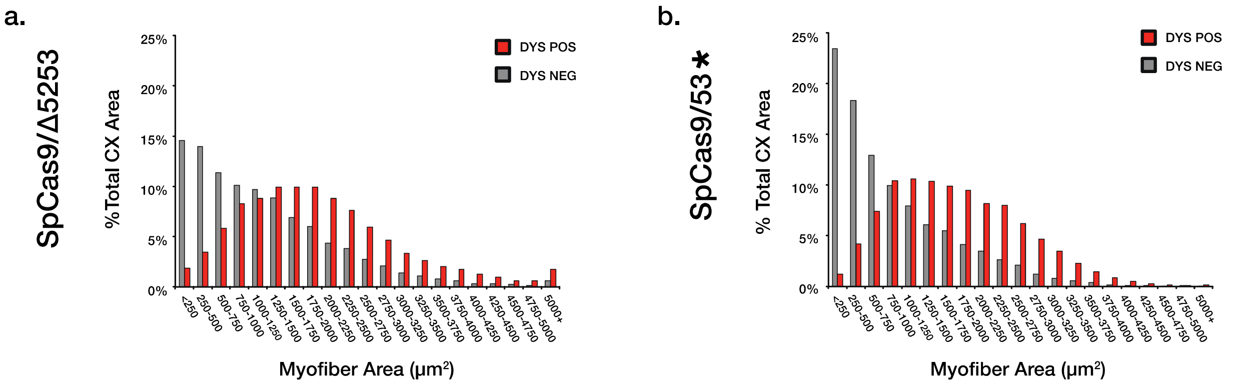


**Supplementary Figure 6: Size distribution analysis for individual myofibers in treated TA muscles.** Cross-sectional area of individual, dystrophin- positive and negative myofibers from transduced TA muscles (n=4 (Δ5253), n=5 (53*) muscles; >25,000 myofibers traced per treatment). Transduced myofibers expressing dystrophin were larger than degenerating dystrophin-negative myofibers following IM injection (**a** and **b**), with a 5- and 10-fold reduction in myofibers under 250 µm$^2$, respectively.

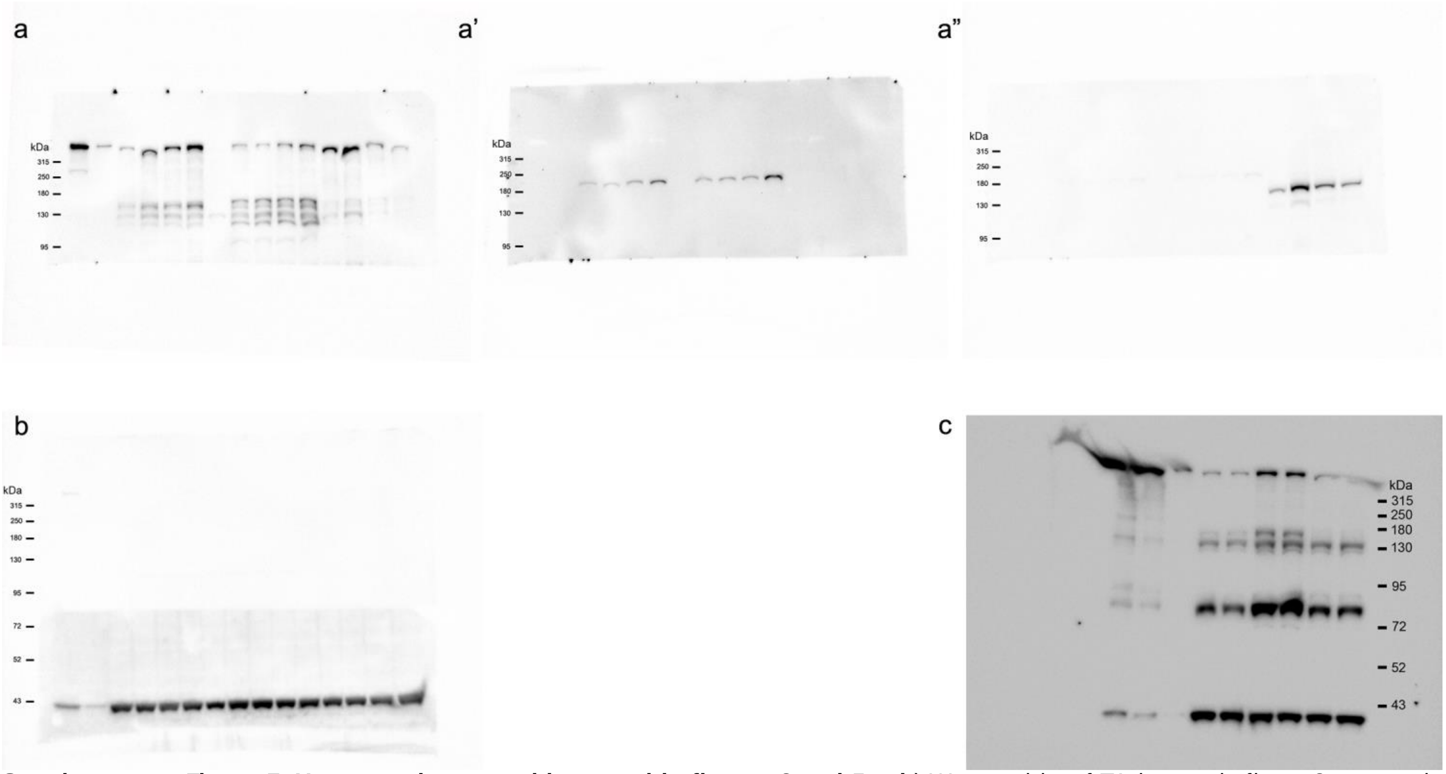


**Supplementary Figure 7: Uncropped western blots used in figures 3 and 5. a-b**) Western blot of TA lysates in figure 3 was cut in half and the top part was stained with antibodies against dystrophin (**a**), SpCas9 (**a'**) or HA-SaCas9 (**a"**). This blot was stripped between each subsequent antibody staining and some residual signal from the SpCas9 staining can be seen in the HA-SaCas9 blot in a". The bottom part of the blot was stained for GAPDH (**b**). **c**) Western blot of cardiac lysates following systemic gene editing was cut in half and stained with antibodies against dystrophin (top half, >72 kDa), or GAPDH (bottom half, <72 kDa).

| ON Target: | sgRNA sequence (5'-3') | Chromosome | Position | Total reads | NHEJ | HDR | NHEJ/HDR (mixed) | Editing efficiency (%) | HDR (%) |
|---|---|---|---|---|---|---|---|---|---|
| **DNA:** | | | | | | | | | |
| (Sp) i51 | GATACTAGGGTGGCAAATAG | X | 84530675-84530694 | 387126 | 33348 | 0 | 0 | 8.61 | 0.00 |
| (Sp) i53 | GTGTTCTTAAAAGAATGGTG | X | 84576353-84576372 | 383505 | 31411 | 0 | 0 | 8.19 | 0.00 |
| (Sa) i51 | GATACTAGGGTGGCAAATAGA | X | 84530675-84530695 | 448016 | 15533 | 0 | 0 | 3.47 | 0.00 |
| (Sa) i53 | GAGATAAATCCCTGCTTATCAC | X | 84576316-84576337 | 870140 | 23263 | 0 | 0 | 2.67 | 0.00 |
| (Sp) 53*-5' (combined w. 3') | (G)TCAAGAACAGCTGCAGAAC | X | 84575591-84575609 | 4681379 | 96177 | 8507 | 1421 | 2.27 | 0.18 |
| (Sp) 53*-3' (combined w. 5') | (G)CAGTTGAATGAAATGTTAA | X | 84575619-84595637 | | | | | | |
| **RNA:** | | | | | | | | | |
| (Sp) 53* (Treated, combined) | | | | 336095 | 22245 | 2692 | 5944 | 9.19 | 0.80 |
| (Sp) 53* (Control, combined) | | | | 486042 | 1292 | 0 | 26 | 0.27 | 0.00 |

**Supplementary Table 1: Deep sequencing quantification of editing efficiency and HDR events using CRISPResso.** Efficient targeting was observed at all target sites for the different approaches. For NHEJ events, the majority of edited reads corresponded to deletions followed by insertions and substitutions (supplementary Fig. 1 & 2). For strategy 2, on-target deep sequencing was performed on DNA and cDNA generated from RNA isolated from treated muscles. Comparing DNA to RNA revealed an increase in both editing efficiency and prevalence of reads corresponding to successful HDR events at the transcript level, likely due to protection of functional edited transcripts against nonsense mediated decay. The sequence used to detect HDR events using CRISPResso included the WT cytosine at the site of the point mutation and both PAM site mutations. HDR quantification does not include mixed NHEJ/HDR events.

| DNA HDR genotypes: Query sequence | Genotype | Reads detected (treated) | % of unaltered reads (Treated) | Reading frame |
|---|---|---|---|---|
| CAAGAACAGCTGCAGAACAGGAGATAACAGTTGAATGAAATGTTAAAGGATTCAACACAA | mdx | 2532317 | 100 | Unaltered in frame |
| CAAGAACAGCTGCAGAACAGGAGACAACAGTTGAATGAAATGTTAAAGGATTCAACACAA | WT | 2294 | 0.091 | In frame |
| CAAGAACAGCTGCAGAACAGAAGACAACAGTTGAATGAAATGTTAAAAGATTCAACACAA | HDR | 4714 | 0.186 | In frame |
| CAAGAACAGCTGCAGAACAGAAGACAACAGTTGAATGAAATGTTAAAGGATTCAACACAA | 5'-pHDR | 295 | 0.012 | In frame |
| CAAGAACAGCTGCAGAACAGGAGACAACAGTTGAATGAAATGTTAAAAGATTCAACACAA | 3'-pHDR | 17 | 0.001 | In frame |
| *Selected single substitutions (background)* | | | | |
| CAAGAACAGCTGCAGAACAGGAGAGAACAGTTGAATGAAATGTTAAAGGATTCAACACAA | N/A | 852 | 0.034 | In frame |
| CAAGAACAGCTGCAGAACAGGAGAAAACAGTTGAATGAAATGTTAAAGGATTCAACACAA | N/A | 325 | 0.013 | In frame |
| CAAGAACAGCTGCAGAACAGCAGATAACAGTTGAATGAAATGTTAAAGGATTCAACACAA | N/A | 211 | 0.008 | In frame |
| CAAGAACAGCTGCAGAACAGTAGATAACAGTTGAATGAAATGTTAAAGGATTCAACACAA | N/A | 766 | 0.030 | In frame |
| CAAGAACAGCTGCAGAACAGGAGATAACAGTTGAATGAAATGTTAAACGATTCAACACAA | N/A | 119 | 0.005 | In frame |
| CAAGAACAGCTGCAGAACAGGAGATAACAGTTGAATGAAATGTTAAATGATTCAACACAA | N/A | 743 | 0.029 | In frame |

| DNA pΔ53 (w/o STOP) genotypes: Query sequence | Deletion size (bp) | Reads detected (Treated) | % of unaltered reads (Treated) | Reading frame |
|---|---|---|---|---|
| CAAGAACAGCTGCAGAACAGGAGA(TAA)CAGTTGAATGAAATGTTAAAGGATTCAACACAA | 0 | 2532317 | 100 | Unaltered in frame |
| CAAGAACAGCTGCAGAACAGGAGACAGTTGAATGAAATGTTAAAGGATTCAACACAA | 3 | 1 | 0.000 | In frame |
| CAAGAACAGCTGCAGAACAGGCAGTTGAATGAAATGTTAAAGGATTCAACACAA | 6 | 0 | 0.000 | In frame |
| CAAGAACAGCTGCAGAACCAGTTGAATGAAATGTTAAAGGATTCAACACAA | 9 | 0 | 0.000 | In frame |
| CAAGAACAGCTGCAGCAGTTGAATGAAATGTTAAAGGATTCAACACAA | 12 | 1 | 0.000 | In frame |
| CAAGAACAGCTGCAGAACAGGGAAATGTTAAAGGATTCAACACAA | 15 | 0 | 0.000 | In frame |
| CAAGAACAGCTGCAGAACAGGAGAATGTTAAAGGATTCAACACAA | 15 | 0 | 0.000 | In frame |
| CAAGAACAGCTGCAGAACAGGATGTTAAAGGATTCAACACAA | 18 | 0 | 0.000 | In frame |
| CAAGAACAGCTGCAGAACAGGAGATTAAAGGATTCAACACAA | 18 | 44 | 0.002 | In frame |
| CAAGAACAGCTGCAGAACATGTTAAAGGATTCAACACAA | 21 | 0 | 0.000 | In frame |
| CAAGAACAGCTGCAGAACAGGTTAAAGGATTCAACACAA | 21 | 2 | 0.000 | In frame |
| CAAGAACAGCTGCAGATGTTAAAGGATTCAACACAA | 24 | 2 | 0.000 | In frame |
| CAAGAACAGCTGCAGAACTTAAAGGATTCAACACAA | 24 | 50 | 0.002 | In frame |
| CAAGAACAGCTGCAGAACAAGGATTCAACACAA | 27 | 1 | 0.000 | In frame |
| CAAGAACAGCTGCAGTTAAAGGATTCAACACAA | 27 | 901 | 0.036 | In frame |
| CAAGAACAGCTGTTAAAGGATTCAACACAA | 30 | 14 | 0.001 | In frame |
| CAAGAACAGCTGCAGAAGGATTCAACACAA | 30 | 55 | 0.002 | In frame |
| CAAGAACAGCTGCAGGATTCAACACAA | 33 | 14 | 0.001 | In frame |
| CAAGAACAGCTGAAGGATTCAACACAA | 33 | 1 | 0.000 | In frame |
| CAAGAACAGAAGGATTCAACACAA | 36 | 0 | 0.000 | In frame |
| CAAGAACAGCTGGATTCAACACAA | 36 | 4 | 0.000 | In frame |
| *Predicted cleavage site deletion product (PAM -3 bp)* | | | | |
| CAGCTGCAGTAAAGGATTCAACA | 28 | 1243 | 0.049 | Out of frame |

Exon 53, PAM, mdx (T) mutation, Substitutions

**Supplementary Table 2: Manual genotype analysis of genomic DNA for strategy 2**. Manual analysis of deep sequencing reads within the Fastq file for on-target PCR amplicons generated from DNA isolated from muscles treated according to strategy 2 (53*). **(Top)** Prevalence of deep sequencing reads corresponding to different HDR derived genotypes and random single nucleotide substitutions (proposed background) at sites of particular interest. (**Bottom**) Prevalence of selected sequences corresponding to partial in-frame deletions (pΔ53) resulting in the removal of the $mdx^{4cv}$ stop codon. The 28 bp out-of-frame deletion sequence predicted to be most prevalent, resulting from DNA cleavage at the prototypical PAM -3 nucleotide position, is indeed found most frequently among the deletions followed by a 27 bp in-frame deletion sequence.

| RNA HDR genotypes: Query sequence | Genotype | Reads detected | | % of unaltered reads | | Reading frame |
|---|---|---|---|---|---|---|
| | | mdx control | Treated | mdx control | Treated | |
| ATCGAATTGAAAGAATTCAGATTCAGTGGGATGAGGTTCAAGAACAGCTGCAGAACAGGAGATAACAGTTGAATGAAATGTTAAAGGATTC | mdx | 242375 | 154693 | 100 | 100 | Unaltered in frame |
| ATCGAATTGAAAGAATTCAGATTCAGTGGGATGAGGTTCAAGAACAGCTGCAGAACAGGAGACAACAGTTGAATGAAATGTTAAAGGATTC | WT | 104 | 45 | 0.043 | 0.029 | In frame |
| ATCGAATTGAAAGAATTCAGATTCAGTGGGATGAGGTTCAAGAACAGCTGCAGAACAGAAGACAACAGTTGAATGAAATGTTAAAAGATTC | HDR | 0 | 1130 | 0.000 | 0.730 | In frame |
| ATCGAATTGAAAGAATTCAGATTCAGTGGGATGAGGTTCAAGAACAGCTGCAGAACAGAAGACAACAGTTGAATGAAATGTTAAAGGATTC | 5'-pHDR | 0 | 61 | 0.000 | 0.039 | In frame |
| ATCGAATTGAAAGAATTCAGATTCAGTGGGATGAGGTTCAAGAACAGCTGCAGAACAGGAGACAACAGTTGAATGAAATGTTAAAAGATTC | 3'-pHDR | 0 | 7 | 0.000 | 0.005 | In frame |
| *Selected single substitutions (background)* | | | | | | |
| ATCGAATTGAAAGAATTCAGATTCAGTGGGATGAGGTTCAAGAACAGCTGCAGAACAGGAGAGAACAGTTGAATGAAATGTTAAAGGATTC | N/A | 24 | 17 | 0.010 | 0.011 | In frame |
| ATCGAATTGAAAGAATTCAGATTCAGTGGGATGAGGTTCAAGAACAGCTGCAGAACAGGAGAAAACAGTTGAATGAAATGTTAAAGGATTC | N/A | 20 | 5 | 0.008 | 0.003 | In frame |
| ATCGAATTGAAAGAATTCAGATTCAGTGGGATGAGGTTCAAGAACAGCTGCAGAACAGCAGATAACAGTTGAATGAAATGTTAAAGGATTC | N/A | 21 | 4 | 0.009 | 0.003 | In frame |
| ATCGAATTGAAAGAATTCAGATTCAGTGGGATGAGGTTCAAGAACAGCTGCAGAACAGTAGATAACAGTTGAATGAAATGTTAAAGGATTC | N/A | 83 | 49 | 0.034 | 0.032 | In frame |
| ATCGAATTGAAAGAATTCAGATTCAGTGGGATGAGGTTCAAGAACAGCTGCAGAACAGGAGATAACAGTTGAATGAAATGTTAAACGATTC | N/A | 17 | 3 | 0.007 | 0.002 | In frame |
| ATCGAATTGAAAGAATTCAGATTCAGTGGGATGAGGTTCAAGAACAGCTGCAGAACAGGAGATAACAGTTGAATGAAATGTTAAATGATTC | N/A | 93 | 51 | 0.038 | 0.033 | In frame |

| RNA pΔ53 (w/o STOP) genotypes: Query sequence | Deletion size (bp) | Reads detected | | % of unaltered reads | | Reading frame |
|---|---|---|---|---|---|---|
| | | mdx control | Treated | mdx control | Treated | |
| ATCGAATTGAAAGAATTCAGATTCAGTGGGATGAGGTTCAAGAACAGCTGCAGAACAGGAGA(TAA)CAGTTGAATGAAATGTTAAAGGATTC | 0 | 242375 | 154693 | 100 | 100 | Unaltered in frame |
| ATCGAATTGAAAGAATTCAGATTCAGTGGGATGAGGTTCAAGAACAGCTGCAGAACAGGAGACAGTTGAATGAAATGTTAAAGGATTC | 3 | 0 | 0 | 0.000 | 0.000 | In frame |
| ATCGAATTGAAAGAATTCAGATTCAGTGGGATGAGGTTCAAGAACAGCTGCAGAACAGGCAGTTGAATGAAATGTTAAAGGATTC | 6 | 0 | 0 | 0.000 | 0.000 | In frame |
| ATCGAATTGAAAGAATTCAGATTCAGTGGGATGAGGTTCAAGAACAGCTGCAGAACCAGTTGAATGAAATGTTAAAGGATTC | 9 | 0 | 0 | 0.000 | 0.000 | In frame |
| ATCGAATTGAAAGAATTCAGATTCAGTGGGATGAGGTTCAAGAACAGCTGCAGCAGTTGAATGAAATGTTAAAGGATTC | 12 | 0 | 0 | 0.000 | 0.000 | In frame |
| ATCGAATTGAAAGAATTCAGATTCAGTGGGATGAGGTTCAAGAACAGCTGCAGAACAGGGAAATGTTAAAGGATTC | 15 | 0 | 0 | 0.000 | 0.000 | In frame |
| ATCGAATTGAAAGAATTCAGATTCAGTGGGATGAGGTTCAAGAACAGCTGCAGAACAGGAGAATGTTAAAGGATTC | 15 | 0 | 0 | 0.000 | 0.000 | In frame |
| ATCGAATTGAAAGAATTCAGATTCAGTGGGATGAGGTTCAAGAACAGCTGCAGAACAGGATGTTAAAGGATTC | 18 | 0 | 0 | 0.000 | 0.000 | In frame |
| ATCGAATTGAAAGAATTCAGATTCAGTGGGATGAGGTTCAAGAACAGCTGCAGAACAGGAGATTAAAGGATTC | 18 | 0 | 87 | 0.000 | 0.056 | In frame |
| ATCGAATTGAAAGAATTCAGATTCAGTGGGATGAGGTTCAAGAACAGCTGCAGAACATGTTAAAGGATTC | 21 | 0 | 0 | 0.000 | 0.000 | In frame |
| ATCGAATTGAAAGAATTCAGATTCAGTGGGATGAGGTTCAAGAACAGCTGCAGAACAGGTTAAAGGATTC | 21 | 0 | 2 | 0.000 | 0.001 | In frame |
| ATCGAATTGAAAGAATTCAGATTCAGTGGGATGAGGTTCAAGAACAGCTGCAGATGTTAAAGGATTC | 24 | 0 | 7 | 0.000 | 0.005 | In frame |
| ATCGAATTGAAAGAATTCAGATTCAGTGGGATGAGGTTCAAGAACAGCTGCAGAACTTAAAGGATTC | 24 | 0 | 42 | 0.000 | 0.027 | In frame |
| ATCGAATTGAAAGAATTCAGATTCAGTGGGATGAGGTTCAAGAACAGCTGCAGAACAAGGATTC | 27 | 0 | 6 | 0.000 | 0.004 | In frame |
| ATCGAATTGAAAGAATTCAGATTCAGTGGGATGAGGTTCAAGAACAGCTGCAGTTAAAGGATTC | 27 | 2 | 1253 | 0.001 | 0.810 | In frame |
| ATCGAATTGAAAGAATTCAGATTCAGTGGGATGAGGTTCAAGAACAGCTGTTAAAGGATTC | 30 | 0 | 35 | 0.000 | 0.023 | In frame |
| ATCGAATTGAAAGAATTCAGATTCAGTGGGATGAGGTTCAAGAACAGCTGCAGAAGGATTC | 30 | 0 | 64 | 0.000 | 0.041 | In frame |
| ATCGAATTGAAAGAATTCAGATTCAGTGGGATGAGGTTCAAGAACAGCTGCAGGATTC | 33 | 0 | 19 | 0.000 | 0.012 | In frame |
| ATCGAATTGAAAGAATTCAGATTCAGTGGGATGAGGTTCAAGAACAGCTGAAGGATTC | 33 | 0 | 0 | 0.000 | 0.000 | In frame |
| ATCGAATTGAAAGAATTCAGATTCAGTGGGATGAGGTTCAAGAACAGAAGGATTC | 36 | 0 | 2 | 0.000 | 0.001 | In frame |
| ATCGAATTGAAAGAATTCAGATTCAGTGGGATGAGGTTCAAGAACAGCTGGATTC | 36 | 0 | 2 | 0.000 | 0.001 | In frame |
| *Predicted cleavage site deletion product (PAM -3 bp)* | | | | | | |
| ATCGAATTGAAAGAATTCAGATTCAGTGGGATGAGGTTCAAGAACAGCTGCAGTAAAGGATTC | 28 | 0 | 582 | 0.000 | 0.376 | Out of frame |

Exon 52, Exon 53, PAM, mdx (T) mutation, Substitutions

**Supplementary Table 3: Manual genotype analysis of transcripts for strategy 2.** Manual analysis of deep sequencing reads within the Fastq files for on-target RT-PCR amplicons generated from RNA isolated from untreated- (mdx control) and treated muscles according to strategy 2 (53*). (**Top**) Prevalence of deep sequencing reads corresponding to different HDR derived genotypes and random single nucleotide substitutions (proposed background) at sites of particular interest. The presence of reads with single nucleotide substitutions within the untreated sample indicates the level of natural variation and/or sequencing errors. Interestingly, inclusion of 2 or more substitutions at defined positions in the query virtually eliminates detection in untreated controls. (**Bottom**) Prevalence of selected sequences corresponding to partial in-frame deletions (pΔ53) resulting in the removal of the *mdx*$^{4cv}$ stop codon. The sequence generated following removal of 27 bp between the two target sites appears to be the most prevalent. The 28 bp out-of-frame deletion sequence, predicted to be most prevalent following cleavage at the prototypical PAM -3 nucleotide position, appears less prominent at the transcript level then at the DNA level (as shown in supplementary table 2).

| OFF Target: | sgRNA sequence (5'-3') | Chromosome | Position | Total reads | Modified Reads | Editing efficiency (%) |
|---|---|---|---|---|---|---|
| **(Sp) sgRNAi51** | GATACTAGGGTGGCAAATAG | | | | | |
| OT#1 | GATACTAGTGTGGCTCATAG | 3 | 141701844-141701863 | 552579 | 1068 | 0.19 |
| OT#2 | GATACGATGGTGGCAAATCG | 7 | 130395430-130395449 | 402732 | 1146 | 0.28 |
| OT#3 | GATACTAGGGTGGGGAATAA | 18 | 39792989-39793008 | 517898 | 2297 | 0.44 |
| **(Sp) sgRNAi53** | GTGTTCTTAAAAGAATGGTG | | | | | |
| OT#1 | TTTTTCTTAAAAGAATGGTA | 17 | 5033249-5033268 | 287696 | 1689 | 0.59 |
| OT#2 | TTGATCTTAGAAGAATGGTG | X | 164639812-164639831 | 48548 | 178 | 0.37 |
| OT#3 | GTTTTCTTGAAAAAATGGTG | 17 | 86877921-86877940 | 406556 | 2047 | 0.50 |
| OT#4 | CTGTTCTTAAAAGGTTGGTG | 4 | 85991645-85991664 | 435323 | 2702 | 0.62 |
| OT#5 | GAGTTCTTCAAAGAATAGTG | 2 | 64505959-64505978 | 485078 | 1653 | 0.34 |
| **(Sp) sgRNA-5'** | (G)TCAAGAACAGCTGCAGAAC | | | | | |
| OT#1 | TCTAGGGCAGCTGCAGAAC | 14 | 11161529-11161547 | 757561 | 2729 | 0.36 |
| OT#2 | TCATTCACAGCTGCAGAAC | 2 | 30938892-30938910 | 347367 | 949 | 0.27 |
| OT#3 | CAAAGAATAGCTGCAGAAC | 11 | 58117430-58117448 | 421793 | 873 | 0.21 |
| OT#4 | TCAAGAACAGCTGCAGCAG | 15 | 3176773-3176791 | 370386 | 2396 | 0.65 |
| OT#5 | TCAAGAACAGCTGCATCAC | 18 | 27855793-27855811 | 459555 | 1846 | 0.40 |
| **(Sp) sgRNA-3'** | (G)CAGTTGAATGAAATGTTAA | | | | | |
| OT#1 | CAGTTACATGAAATGTTAA | 3 | 106429607-106429625 | 430282 | 1261 | 0.29 |
| OT#2 | CATTTTAATGAAATGTTAA | 7 | 86738658-86738676 | 495137 | 1978 | 0.40 |
| OT#3 | AAGTTGAATGAAATTTTAA | 5 | 63409984-63410002 | 275293 | 1531 | 0.56 |
| OT#4 | CAGTGGAATAAAATGTTAA | 2 | 23248164-23248182 | 145528 | 1369 | 0.94 |

**Supplementary Table 4: Genomic off-target analyses.** Deep sequencing quantification of gene editing frequency at the top predicted potential off-target sites for each gRNA reveals low levels of sequence variation (nucleotide mismatches from the on-target sequence are highlighted in blue). The vast majority of edited reads detected at potential off-target sites correspond to single nucleotide substitutions. Few deletion- and insertion events are randomly distributed across the amplicons, indicating low levels of natural variation and/or sequencing errors.

**5’-**GTACCTTTCTAATAAATAATTGTTATTTAGTGTCAGAGTCTAAAGTTGAATTTATATTTCTAAACATGGCA
CCAATATTGTAGTTTATTTCAATGCAAGTAATTTAATAGAAAGTCAAATTTGTCACCTGAAGAAATGATTTTGTTAATTATTTTACCTAT
ATCACTCATAGCACCTTGGATATATTTAATGAGAAATATACATGTGCAATGACGTTTAGATTCTAAATTTCCACTGTCTTCTCTTGAGTA
ATAATTACTGTTCTTTATTCTTATTTTTATTCCAGTTGAAAGAATTCAGATTCAGTGGGATGAGGTTCAAGAACAG CTGCAGAACAGAA
GACAACAGTTGAATGAAATGTTAAAAGATTCAACACAATGGCTGGAAGCTAAGGAAGAAGCCGAACAGGTCATAGGACAGGTCAG
AGGCAAGCTTGACTCATGGAAAGAAGGTCCTCACACAGTAGATGCAATCCAAAAGAAGATCACAGAAACCAAGGTTAGTGTCAAGC
ATATCTTTAAAAAAATATTTTGTATAGCAAATGAAAGCATGCCATAAATTAAAATTTAATGTTTTCTTAGTG AAAATTACATTTAGGAA
GTGAAAAGTGGAATTCTTGCTTGTTTTTGATTGGTTGGTTTGTTGGTTGGTTGGTTGGTTGGCTGGCTGGTTGGTTGGTTGGCTGGTT
GGTTGGTTGGTTGGTTTTGAGACAAAAATCTAAACTCAAAATACTCAAGACTACAGATGAGTGCCACTACATCTACATGATTTAAAAT
TTTGAGACACAGTATAGGTTATAGGAAAACTG**-3’**

**Supplementary Table 5**: **Sequence of the HDR fragment used in the AAV vectors shown in Figure 1c.** The silent PAM site mutations that were introduced to prevent vector cleavage by Cas9 (G to A) are marked in blue. The wild type nucleotide (C) used for replacement of the *mdx*$^{4cv}$ mutation (T) is marked in green.

| | | | |
|---|---|---|---|
| **SpCas9 subcloning** | | | |
| Forward | ATGGCCCCAAAGAAGAAGCG | Reverse | CTTACTTTTTCTTTTTTGCCT |
| **U6-(Sp)sgRNA cassette subcloning** | | | |
| Forward | TCAGACCCACCTCCCAAC | Reverse | AATTCAAAAAAGCACCGACTCG |
| **U6-(Sa)sgRNA subcloning** | | | |
| Forward | TCAGACCCACCTCCCAAC | Reverse | AATTCAAAAAAGCACCGACTCG |
| **sgRNA primers for Strategy A (Δ5253)** | | | |
| SpCas9-intron 51 (Forward) | CACCGATACTAGGGTGGCAAATAG | SpCas9-intron 51 (Reverse) | AAACCTATTTGCCACCCTAGTATC |
| SpCas9- intron 53 (Forward) | CACCGTGTTCTTAAAAGAATGGTG | SpCas9- intron 53 (Reverse) | AAACCACCATTCTTTTAAGAACAC |
| SaCas9- intron 51 (Forward) | CACCGATACTAGGGTGGCAAATAGA | SaCas9- intron 51 (Reverse) | AAACTCTATTTGCCACCCTAGTATC |
| SaCas9- intron 53 (Forward) | CACCGAGATAAATCCCTGCTTATCAC | SaCas9- intron 53 (Reverse) | AAACGTGATAAGCAGGGATTTATCTC |
| **sgRNA primers for Strategy B (53*), (preferred G inserted at 5' position)** | | | |
| SpCas9-5' (Forward) | CACCGTCAAGAACAGCTGCAGAAC | SpCas9-5' (Reverse) | AAACGTTCTGCAGCTGTTCTTGAC |
| SpCas9-3' (Forward) | CACCGCAGTTGAATGAAATGTTAA | SpCas9-3' (Reverse) | AAACTTAACATTTCATTCAACTGC |
| **On-target PCR/RT-PCR primers** | | | |
| PCR-Δ5253 (Forward) | CTCATACCCAAAGCTGCTAG | PCR-Δ5253 (Reverse) | CCTTTTAGCCTAGAGAGTGC |
| PCR-53* (Forward) | GCTGAGGTAATAGAGCCAAG | PCR-53* (Reverse) | CTGTGATCTTCTTTTGGATTGC |
| RT-PCR_Δ5253 (Forward) | GCCATCTTCTTTGCTGTTGG | RT-PCR_Δ5253 (Reverse) | TCCCGAAGAAGTTTCAGTGC |
| RT-PCR_53* (Forward) | CAGAGAGTGATGGTGGGTGA | RT-PCR_53* (Reverse) | TCCCGAAGAAGTTTCAGTGC |
| **On-target deep sequencing primers** | | | |
| DS_i51 (Forward) | CACACATTTGTCCTTATGATTAAGATTTGG | DS_i51 (Reverse) | GCAACAACACTCTTAAACACTGAG |
| DS_i53 (Forward) | ATGTCCTTTGCCACCATGCTAA | DS_i53 (Reverse) | GCATCTATTACTTTTCCTAAGAAGAAATT |
| DS_53* (Forward) | TTTCCACTGTCTTCTCTTGAGTAA | DS_53* (Reverse) | CTACTGTGTGAGGACCTTCTTTC |
| DS_RT53* (Forward) | CCAGCAATCAAGAAGCTAGAAC | DS_RT53* (Reverse) | GCATCTACTGTGTGAGGACC |
| **Off-target deep sequencing primers** | | | |
| DS_i51OT#1 (Forward) | CTGTTTGCTCATGGGCTCA | DS_i51OT#1 (Reverse) | ACAGTCTTTCATAACAGAATGCT |
| DS_i51OT#2 (Forward) | CCAACGACTGTTTCTGAACC | DS_i51OT#2 (Reverse) | GAAATGTCTCTACCCAGTTTGC |
| DS_i51OT#3 (Forward) | GCCATCCTCAAAGAAGAACCAAG | DS_i51OT#3 (Reverse) | TGGAGCACCACATTTAGCCATC |
| DS_i53OT#1 (Forward) | GATGGCTTTGAACTTGAGAGAG | DS_i53OT#1 (Reverse) | CTAACACACTCGGGGAACTG |
| DS_i53OT#2 (Forward) | GAGTTGATTCTCCTTCCACATC | DS_i53OT#2 (Reverse) | TTCGTTCCACCTACCAGAAG |
| DS_i53OT#3 (Forward) | AGCCTTAGCGTTGGTACAG | DS_i53OT#3 (Reverse) | ATTCATTACCTCATAGTTATGCTGCT |
| DS_i53OT#4 (Forward) | CCTGTAACCAGACAAGACTTCTAG | DS_i53OT#4 (Reverse) | GTCACTGGTAGTTAATAGGGTACG |
| DS_i53OT#5 (Forward) | CACCTGTTGACGACAGTAACAA | DS_i53OT#5 (Reverse) | AGTCAAATGACAAGACTATGGCTAC |
| DS_53*-5'OT#1 (Forward) | CGAGCCACAGTGAATTGATG | DS_53*-5'OT#1 (Reverse) | CGTTCCATAGACAAGTGTCAG |
| DS_53*-5'OT#2 (Forward) | GCTGCCTGGGAGGTTTAGA | DS_53*-5'OT#2 (Reverse) | GGTTCAGCCTCTCAGTGGG |
| DS_53*-5'OT#3 (Forward) | CAATGGAGCAGAAAACGTCTG | DS_53*-5'OT#3 (Reverse) | GCACAGTCCACATCCTCATTC |
| DS_53*-5'OT#4 (Forward) | GGCTTGGTTCACTTTTCCACTC | DS_53*-5'OT#4 (Reverse) | CTCAGTGGCAGTTGAATACCAG |
| DS_53*-5'OT#5 (Forward) | GTCTCAGTCTCAGTGAACAAGTG | DS_53*-5'OT#5 (Reverse) | GGCACAGCATGTACTAGAGAT |
| DS_53*-3'OT#1 (Forward) | AGCAGGGTTCGCTTGTAG | DS_53*-3'OT#1 (Reverse) | GCTCACTTGACTTTGAAGGC |
| DS_53*-3'OT#2 (Forward) | AGCATGGACAATGAGGAACT | DS_53*-3'OT#2 (Reverse) | CCTGGGTGTTTTGCTTTTCT |
| DS_53*-3'OT#3 (Forward) | TACAGGTGAGTTGACACACTCC | DS_53*-3'OT#3 (Reverse) | AGGATTTCAGCATCACTATCTAGC |
| DS_53*-3'OT#4 (Forward) | CCTTGCCATTCTGCCTTTTCC | DS_53*-3'OT#4 (Reverse) | CATTCTGTGTGAGGTAACCCAG |

**Supplementary Table 6: List of primers.** Primer pairs used for subcloning; SpCas9 from pSpCas9(BB)-2A-Puro (PX459) (Addgene plasmid# 48139) into the pAAV-CK8-SpCas9 nuclease vector, the U6-(Sp)sgRNA cassette from plasmid lentiCRISPRv1 (Addgene plasmid# 49535) into the pAAV-Δ5253/53* targeting vectors, an additional U6-(Sa)sgRNA cassette into the plasmid pX601-AAV-CMV::NLS-SaCas9-NLS-3xHA-bGHpA;U6::BsaI-sgRNA (Addgene plasmid# 61591), as well as sgRNA target sequences and primers used to amplify ON/OFF target sites for PCR, RT-PCR and deep sequencing (DS) analyses (sequences are shown in the 5'-3' orientation).
