# Supplementary material for: Corrigendum: Muscle-specific CRISPR/Cas9 dystrophin gene editing ameliorates pathophysiology in a mouse model for Duchenne muscular dystrophy
Source: Nat Commun. 2017 Jun 23;8:16007. doi: 10.1038/ncomms16007 (PMC5489999; doi:10.1038/ncomms16007)
Supplement: Supplementary Data 1 [file ncomms16007-s1.pdf]

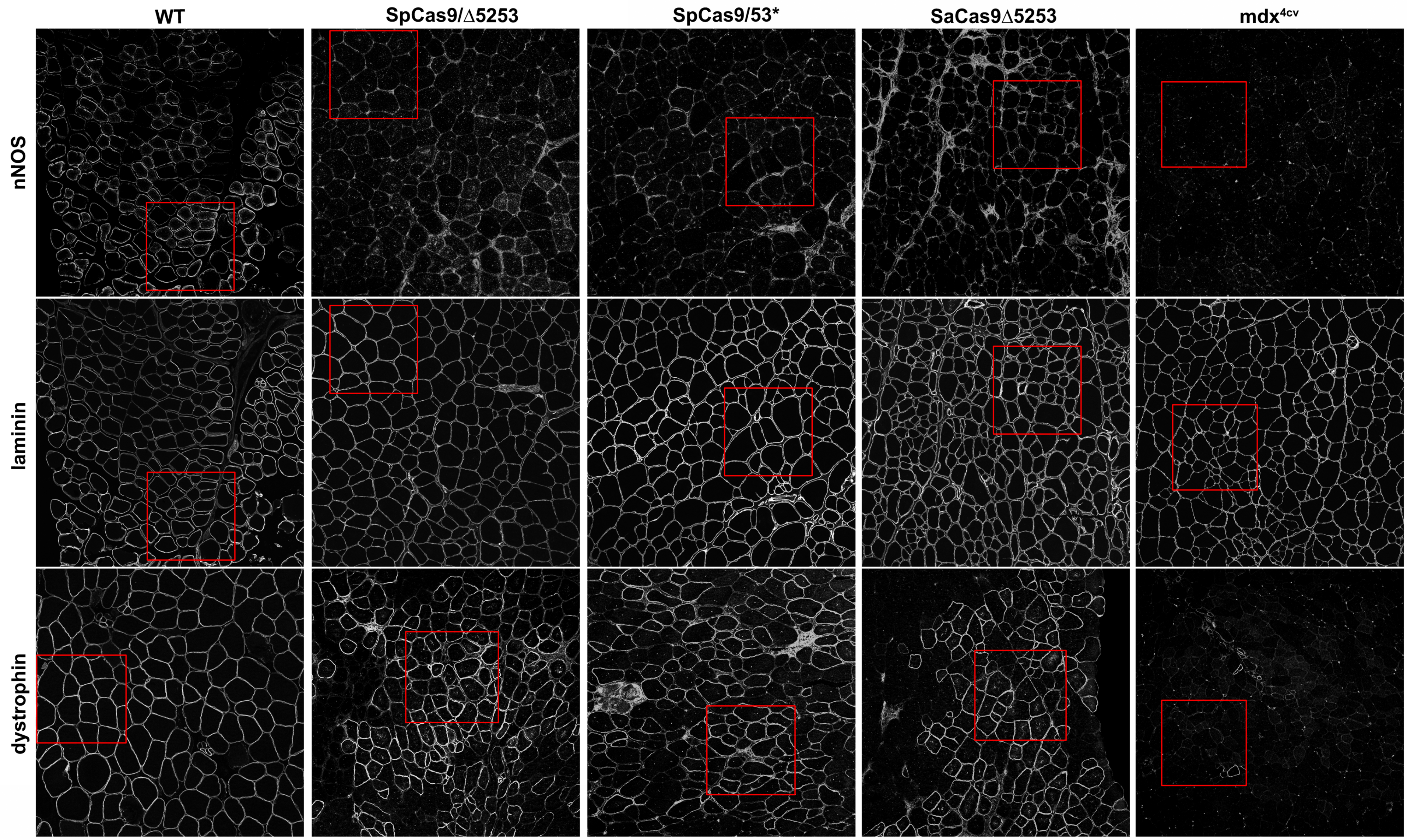

Supplementary Figure 8: Images used to assemble panels in Figure 4a (regions used in panels are outlined in red). TA muscle cross-sections co-stained for nNOS and laminin (top and middle panels), representative sections stained for dystrophin (bottom panels), since both nNOS and dystrophin antibodies were raised in rabbit.
